# Correlative Electron Paramagnetic Resonance Imaging and Atomic Force Microscopy of Lithium Deposited on Copper

**DOI:** 10.1002/cphc.202400937

**Published:** 2025-01-24

**Authors:** Beatrice Wolff, Christian Hellenbrandt, Peter Jakes, Rüdiger‐A. Eichel, Josef Granwehr, Florian Hausen

**Affiliations:** ^1^ Forschungszentrum Jülich GmbH Institute of Energy Technologies, IET-1 52425 Jülich Germany; ^2^ RWTH Aachen University Institute of Physical Chemistry 52074 Aachen Germany; ^3^ RWTH Aachen University Institute of Technical and Macromolecular Chemistry 52074 Aachen Germany

**Keywords:** electron paramagnetic resonance lmaging, atomic force microscopy, correlative microscopy and spectroscopy, anode free batteries, lithium batteries

## Abstract

Anode free concepts are gaining traction in battery research. To improve cyclability, a better understanding of the deposition processes and morphologies is necessary. Correlative experiments enable a link between a variety of properties obtained, such as chemical, mechanical or electrochemical data. Here, electron paramagnetic resonance imaging (EPRI) is correlated with atomic force microscopy (AFM) to gain a deeper understanding of the microscopic topography and local stiffness at different intensities of the lithium selective EPRI map. Experiments were carried out on a sample of lithium deposited on copper foil from standard battery electrolyte. The correlation of both methods reveals that EPRI has a high sensitivity towards small lithium structures, while bulk lithium was not detected. The results demonstrate that EPRI can be used for prescreening to identify regions with different properties, which can then be analysed individually by AFM.

## Graphical abstract

The correlation of a spectroscopic (EPRI) and microscopic (AFM) technique is demonstrated for a sample of Li deposited on Cu. Areas of different morphology were analysed. The techniques complement each other's length scales as well as the information obtained from the sample. In addition to spectroscopic information, we analyse topographical and micromechanical properties of the sample.

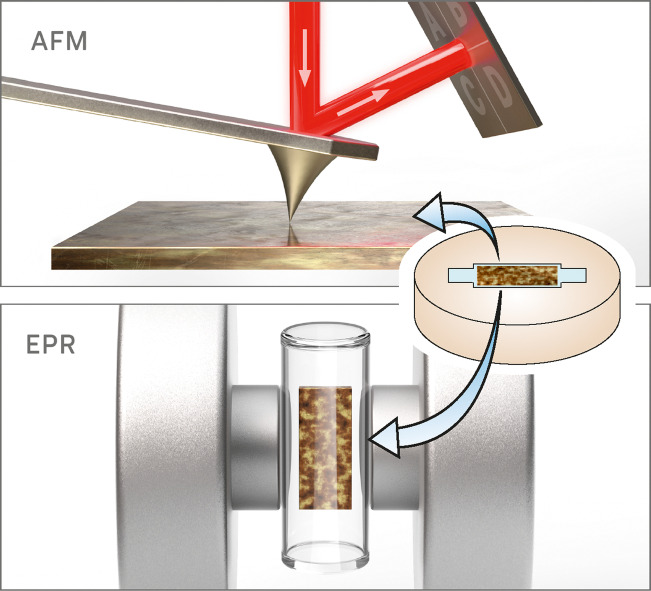

## Introduction

Anode free batteries are intensively researched as concepts for next‐generation batteries and thus, a better understanding of the deposition process, growth mechanisms and morphologies becomes necessary.[^1^, ^2^, ^3^] Both atomic force microscopy (AFM)[^4^, ^5^, ^6^, ^7^, ^8^] and electron paramagnetic resonance imaging (EPRI),[^9^, ^10^, ^11^, ^12^] which is based on electron paramagnetic resonance (EPR) spectra, have provided valuable results for the study of Li deposition and morphologies.

A particularly interesting process for anode free batteries is the electrochemical deposition of Li on Cu, as this is typically used as current collector in Li‐ion batteries (LIBs). The morphology of deposited Li depends on various factors, such as the the current density, with higher current densities leading to a more uniform deposition,[^13^, ^14^, ^15^] or pre‐conditioning of the substrate with nucleation modifiers.[^16^, ^17^] AFM is an invaluable tool to study Li deposition processes on Cu on the microscale. It can unravel the morphology of Li deposited on Cu in different electrolytes *in situ*.[^18^, ^19^, ^20^, ^21^] Furthermore, the effect of electrode surface potential on Li deposition has been analysed.^[22]^

Within this manuscript, a correlative study between EPRI and AFM of Li deposition on Cu is introduced. The aim of this paper is twofold: Firstly, gaining a deeper understanding of the microscopic topography and local stiffness at different intensities of the Li selective EPRI map. As AFM is only capable of providing surface related information, it is correlated with EPR. Secondly, the complex data evaluation and interpretation of conduction EPR and EPRI benefits greatly from the additional information obtained by AFM.

EPR is a spectroscopic technique that is sensitive to paramagnetic species, i. e. species with unpaired electrons.[^23^, ^24^] Considering metallic species, the paramagnetic conduction electrons of light metals, such as Li, can be studied by EPR.^[25]^ In contrast, conduction electrons of Cu are EPR silent. Conduction EPR can often be represented as phase‐shifted Lorentzian lines, containing characteristic information such as the resonance frequency, linewidth and phase, which affects the line shape.^[26]^ The area spanned by the spectrum correlates with the number of spins contributing to the signal. For conduction electrons, this number is related to the density of states at the Fermi level. For conductive samples, EPR probes only the surface and near‐surface region of the sample due to the skin effect.[^10^, ^27^, ^28^] In addition to single EPR spectra considering the entire sample surface, the spin density can be resolved spatially. EPRI records spatial maps of paramagnetic species. It can achieve a resolution in the micrometre regime. This resolution is affected by several factors, amongst others noise and linewidth of the paramagnetic species in question.^[29]^ Here, smaller linewidths facilitate a higher spatial resolution. Typical fields of view are in the order of 1×1 cm^2^. For battery research, EPR is a potent method since it detects Li metal and provides further information on the Li morphology, as the linewidth of the Li EPR signal depends on the Li morphology.^[10]^ This property has been used to study the evolution of mossy Li during cycling.^[30]^ EPRI has been employed in battery research to localise dendrites in a separator^[10]^ and observe Li deposition *in situ*.[^9^, ^12^] However, in metallic and semiconducting layers, the EPR line shape can be distorted, depending on the layer thickness and conductivity.^[31]^ This leads to difficulties in spectral analysis^[10]^ which are currently not fully understood.

AFM is a microscopic, surface sensitive technique that allows one to obtain the three‐dimensional topography of a sample on the microscale as well as probing micromechanical and microelectrical properties.^[32]^ It works by scanning a tip attached to a cantilever across the sample surface. The deflection of the cantilever is recorded. To obtain mechanical data such as the local distribution of stiffness, the AFM tip is indented into the sample surface at every pixel of the image.

As both EPRI and AFM probe different aspects of a sample on different length scales and sampling depths, the correlation of these two methods provides a deeper understanding of relevant processes and phenomena. EPRI profits from the high resolution of AFM as well as the possibility to study all species on the sample surface regardless of their magnetic properties. Furthermore, the possibility to reference different EPR signal shapes with corresponding morphologies is of avail. AFM on the other hand, benefits from the chemical identification of species on a larger scale,^[10]^ as long as the sample contains relevant paramagnetic species. In particular, this allows one to distinguish between metallic Li and other possible deposits on the sample surface.

AFM has previously been used correlatively with a variety of techniques, such as other microscopic methods like scanning electron microscopy[^33^, ^34^] or optical microscopy[^35^, ^36^] as well as spectroscopic techniques such as Raman spectroscopy.^[37]^ EPRI has been correlated to magnetic resonance imaging (MRI)[^38^, ^39^] and optical images^[10]^ but no correlation to microscopic techniques has been reported so far.

In this manuscript, a correlative AFM and EPR imaging study of Li deposition on Cu foil is presented. EPRI, which is sensitive to Li but not to Cu, selectively visualises the Li distribution on a global scale. AFM provides information about the morphology and local stiffness on the microscale. A sample holder suitable for both analytical modalities was designed, which was used to study the same sample using both techniques. Direct correlation of complementary spectroscopic and microscopic information was employed to investigate the homogeneity of Li plating, and to characterise variations of mechanical properties of the plated Li.

## Experimental

Li foil was stored in an argon filled glove box and used as‐received in a thickness of 300 μm (Honjo Metal Co.,Ltd., Japan). Cu foil was also used as‐received in a thickness of 10 μm (Evonik, Germany). The electrolyte consisted of 1.2 M LiPF_6_ dissolved in ethylene carbonate and ethyl methyl carbonate (3 : 7 by weight, Tomiyama, Japan). Sample preparation, transfer to a sealed tube for EPR measurements and AFM measurements were carried out inside an argon filled glove box (O_2_ <0.1 ppm, H_2_O <0.1 ppm, MBraun).

**Figure 1:**
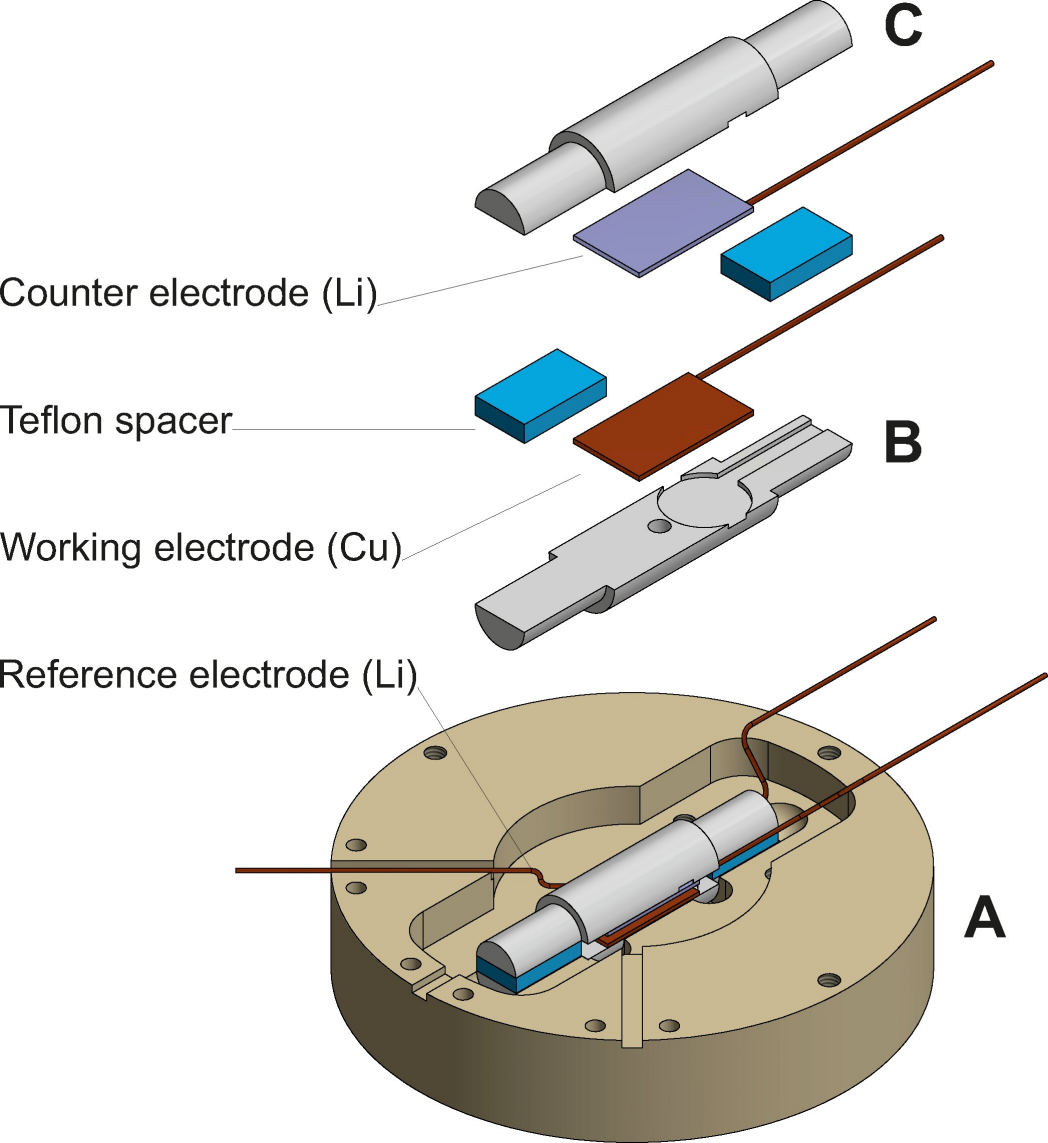
Schematical setup of the custom‐made electrochemistry cell used for sample preparation. It is derived from an electrochemical AFM cell and holds the *operando* EPR cell. A) PEEK body of the electrochemistry cell, B) bottom half‐shell of the EPR cell, C) top half‐shell of the EPR cell.

The sample was prepared in a custom‐made cell. As shown in Figure 1, the cell consists of a polyether ether ketone (PEEK) body (A) that fits a quartz glass cell (B, C). The quartz glass cell is derived from the *operando* EPR cell published elsewhere^[40]^ and is used for the EPR imaging experiments. The PEEK body is derived from typical AFM *operando* cells and has a cavity that fits a half‐shell of the EPR cell. This setup can also be used for *operando* AFM experiments if the counter electrode is mounted in a circle around the working electrode.^[41]^ In this study, deposition of Li on Cu was performed before EPR and AFM experiments inside the EPR sample holder. A parallel plate setup was chosen for Li electrodeposition. To avoid damaging of the sample surface for later AFM experiments, plating was conducted without a separator between the electrodes. Instead, Teflon spacers were fixed between the half‐shells of the quartz‐cell, shown in blue in Figure 1. The spacers provided a distance of 3 mm between the half‐shells. The quartz‐cell was fixed inside the PEEK body and the cell filled with electrolyte. A reference electrode made from Li wrapped around an insulated Cu wire was inserted into the PEEK body next to the quartz glass cell. On the bottom half‐shell (B), a piece of Cu foil was fixed with Kapton tape as working electrode, the exposed area has dimensions of 8×16 mm^2^. On the top half‐shell (C), a piece of Li foil was fixed in the same way as counter electrode.

Li was deposited on the Cu foil electrochemically. The corresponding voltage profile is presented in Figure S1. Deposition on the surface was performed in two steps, the total amount of deposited Li corresponds to a charge of 0.8 mAh/cm^2^. At first, a seeding step was applied with a current density of −20 mA/cm^2^ for 0.3 s. Afterwards, Li was deposited at a current density of −1 mA/cm^2^ for 30 min, corresponding to a charge of 0.5 mAh/cm^2^.

The half‐shell of the quartz glass cell holding the sample (B) was transferred to a 10 mm quartz glass tube which was then sealed inside the glovebox. EPR experiments were carried out on an E540 Elexsys X‐band spectrometer (Bruker) equipped with a 4108 TMHS resonator. The imaging setup as well as image reconstruction have been described elsewhere.^[10]^ The EPR spectrum was recorded with a microwave power of 0.63 mW, a modulation amplitude of 0.1 mT and a modulation frequency of 100 kHz. The sweep width was 10 mT with a sweep time of 123 s, 4096 points were recorded. For the EPR image, the same parameters were used. A gradient of 8 mT/cm was applied. The image was recorded with 512 pixels in a field of view of 10 mm and 402 projections.

**Figure 2:**
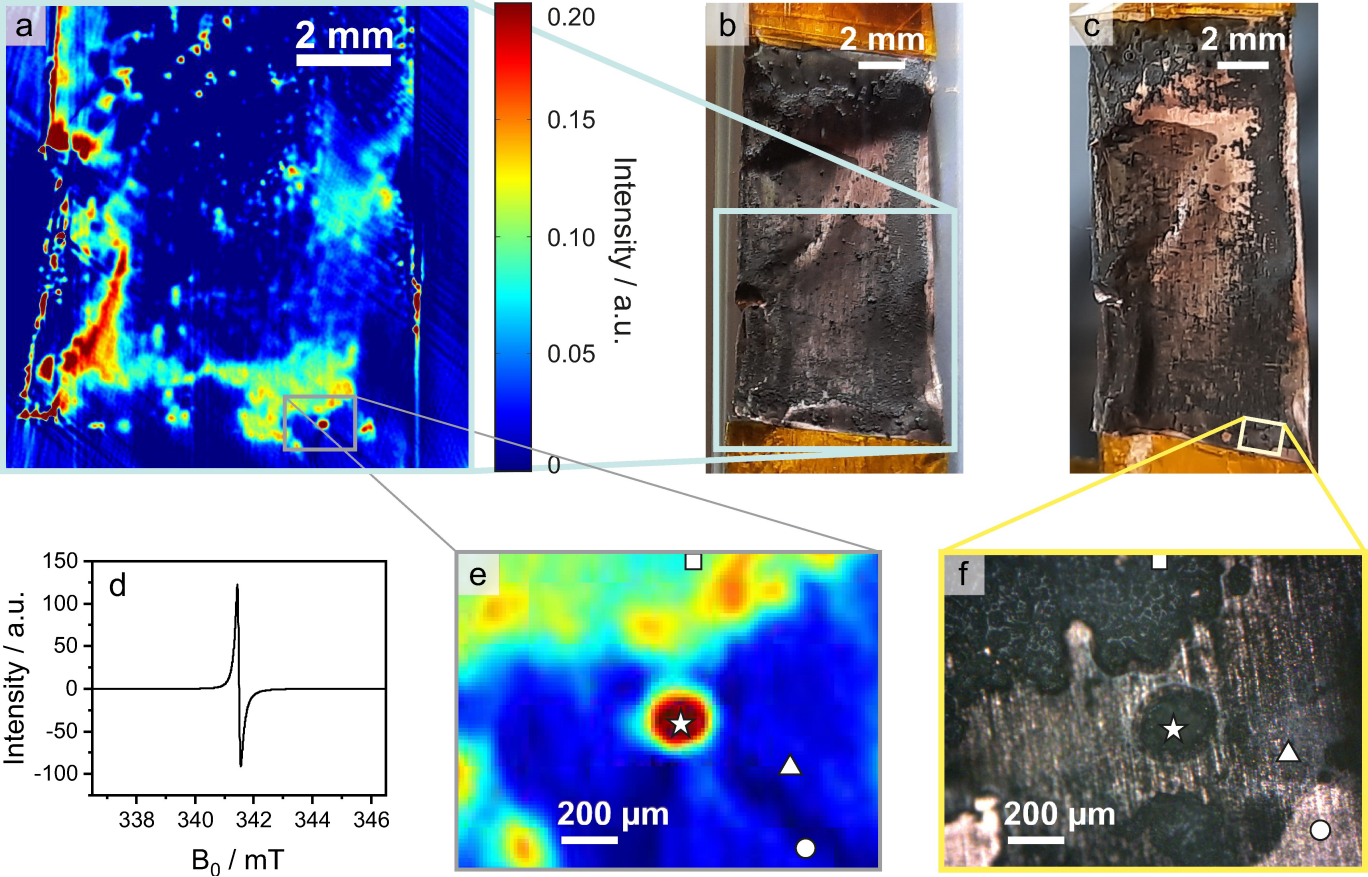
Optical images, EPRl map and EPR spectrum of Li deposited on Cu. a) EPRl map, b) optical image of sample prior to EPR measurement, c) optical image of sample after transfer to AFM, d) EPR spectrum of entire sample surface, e) enlarged section of EPRl map, detailing the positions at which AFM images were recorded, f) optical microscopic image, detailing positions at which AFM images were recorded. The symbols square, star, triangle, circle are used to reference the positions at which AFM images were obtained.

AFM experiments were performed on a Dimension Icon (Bruker) with a boron doped diamond tip (ADAMA, AD‐40‐AS). Images were obtained in PeakForce tapping quantitative nanomechanical mapping (QNM) mode (Bruker, proprietary mode).[^42^, ^43^] In this mode, a force–distance curve is acquired at every pixel of the image. From the force–distance curves, stiffness is determined as reduced modulus by fitting the retracting part of these curves according to the Derjaguin–Muller–Toporov (DMT) model.^[44]^ For tip calibration, the deflection sensitivity was determined from force–distance curves on a hard sapphire sample. The spring constant was then determined as k=52.8 N m^−1^ via thermal tune. The tip radius of 10 nm was determined relatively by modulus determination of a highly oriented pyrolytic graphite and a polystyrene film reference sample (Bruker) with nominal elastic moduli of 18 GPa and 2.7 GPa, respectively. A setpoint of 200 nN was used for images obtained at the position indicated by a circle, cf. Figure 2. All other images were recorded with a setpoint of 150 nN.

## Results and Discussion

Metallic Li was deposited electrochemically on Cu foil. To improve the quality of the deposited Li layer, Li was seeded by depositing Li at a high current density for a short period of time.[^13^, ^14^] Afterwards, Li was deposited at a current density of −1 mA/cm^2^ for 30 min.

Figure 2b shows a photographic image of the sample. It is clear that Li deposits are distributed unevenly on the sample surface. Especially close to the edges of the sample, higher amounts of Li are present. Furthermore, it is found that the deposition occurs both as thin sheet of Li as well as small particles growing on the surface. It should be noted that the Cu foil is not entirely flat. This does, however, not seem to impact the deposition of Li as observed from the optical image.

The EPR spectrum of the sample is depicted in Figure 2d. Here, the entire sample surface contributes to the EPR signal. It exhibits a slightly asymmetric line shape. This generally proves that metallic Li was deposited on the sample, confirming the optical images in which dark depositions are observed. A thick and dense layer of Li exhibits an asymmetric line shape, whereas for mossy and dendritic Li a nearly symmetric line shape has been reported, with a higher symmetry and narrower line width for dendritic Li.^[10]^ The line shape observed here is consistent with previous reports of mossy Li deposits.[^10^, ^30^] Although the optical images show large areas of flat Li deposits, there is no thick bulk Li detected on the sample, which would appear as a broader, asymmetric line shape.

For the more experienced reader, the following paragraph goes deeper into the details of the EPR line shape. While we would have observed a broad asymmetric Dysonian line shape for a thick layer of bulk Li,^[27]^ the line shape observed here is nearly symmetric. The same has also been reported for thin layers of bulk Li with a thickness below the skin depth, which is about 1.1 μm for Li metal at X‐band frequencies.^[45]^ The EPR spectrum shown here was recorded with identical parameters as the subsequent EPRI map and slightly overmodulated to optimise the signal to noise ratio.^[23]^ A reference measurement with a modulation amplitude of 0.01 mT resulted in a line width of 0.06 mT which is broader than expected for mossy Li, when compared to the line width observed by Niemöller *et al*.^[10]^

Figure 2a shows a 2D‐map of the Li distribution on the sample obtained by EPRI, spanning an area of 1×1 cm^2^. This area is marked by a light blue square in Figure 2b. Areas of different signal intensity are identified. High intensities are observed preferentially close to the edges of the sample as seen on the left side of the Cu foil. This correlates with the higher amounts of Li close to the sample edge observed in the optical image of the sample. The overall image is in agreement with the EPRI maps observed by Geng *et al*. for Li deposition on Cu foil from a Li cobalt oxide cathode.^[12]^ Areas of low intensity, depicted in dark blue, correlate to areas of none or very thin Li plating where the Cu foil shines partly through the deposits in the optical image. Areas of medium intensity, depicted in cyan/light blue, indicate thicker Li plating according to the optical image. For areas with high intensity, depicted in yellow to red colours, small protrusions are observed on the surface. The absence of a bulk Li signal in the EPR spectrum indicates that these high intensity signals are caused by thin layers of metallic Li or deposits of porous, mossy Li.

To get a better insight into the morphology of the sample, AFM was performed on selected areas of the sample, marked according to the symbols in Figure 2f. Figure 2c displays an optical image of the sample after it was transferred to the AFM. A few Li protrusions got damaged during transfer and are missing from the bottom right corner of the sample. Figure 2f was obtained by the internal optical microscope of the AFM and illustrates the individual regions of interest further investigated by AFM. Figure 2e depicts the corresponding section of the EPRI map.

Four different spots were chosen to investigate correlatively by AFM, mapping the topography and micromechanical properties. Firstly, a spot at high intensity (dark red) in the EPR image was chosen, marked by a star. In the microscopic image, this spot is part of a nearly circular protrusion of Li deposits on the Cu surface. Secondly, a spot was chosen at medium intensity (cyan blue) of the EPR image, marked by a square. This spot is part of a large area of rather thick Li deposits, appearing as a solid black layer on the image of the optical microscope in Figure 2. The third and fourth spot were both chosen at minimum intensity (dark blue) in the EPR image. In the optical image, however, both spots show slightly different characteristics. The spot marked by a triangle is located in an area of very thin deposits, where the underlying Cu foil barely shines through. The spot marked by a circle is part of an area that displays only very thin and scarcely scattered deposits on the Cu surface, which appears as nearly blank Cu foil in optical images. For comparison, the bottom left corner of Figure 2f demonstrates an area where the Li deposits broke off during transport, showing bare Cu foil. This part is even more shiny than the area on the bottom right corner, where the spot marked by a circle is located. In the following text, the spots marked by the different symbols will be referred to as position square/star/triangle/circle.

**Figure 3:**
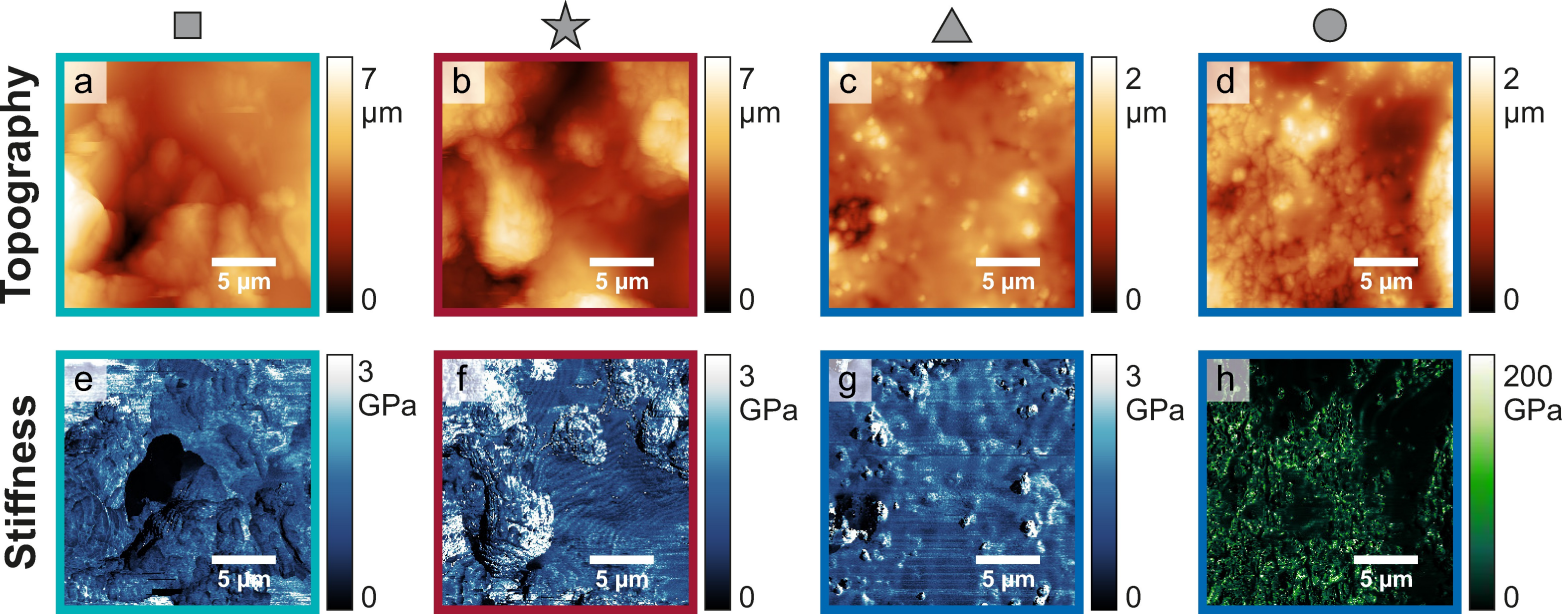
Topography (a–d) and stifness maps (e–h) of Li deposited on Cu. The symbols square/star/triangle/circle mark the position at which the images were obtained on the sample, cf. Figure 2. The coloured boxes refer to the intensity observed at the respective position in the EPRl map.

Figure 3a–d depicts the topographic maps obtained by AFM at the four selected positions of 20×20 μm^2^. At positions square and star (Figure 3a, b), irregularly shaped protrusions with high surface roughness are observed. These are interpreted as “mossy” Li deposits. Comparing positions square and star, the irregularity and number of large protrusions appears to be higher for position star. Positions triangle and circle (Figure 3c, d) both demonstrate a markedly different surface morphology than positions square and star. This difference is also reflected in the surface roughness, presented in Table 1. The surface roughness was calculated as the root mean square (rms) of the height. The higher roughness at positions square and star is in accordance with the interpretation of a mossy Li morphology, whereas the smoother surfaces might hint at bulk Li deposition. The topographical map of position triangle depicts a relatively smooth surface film, with several embedded particles scattered across the surface. At position circle, the topographical map displays a granular surface which is covered by a smooth surface film only in few places. Assuming that the smooth surface film with protruding particles corresponds to Li deposits and the granular surface corresponds to the Cu surface, the coverage of the Cu surface with Li deposits is higher at position triangle compared to position circle. This can also be observed in the optical image of the sample (Figure 2e), as the Cu surface is covered by more dark deposits at position triangle. Nonetheless, there are Li deposits present also at position circle, as indicated by the dark spots scattered across the Cu surface in the optical image and the patches of smooth surface layer on the granular surface in the AFM topography. Although Li and Cu are assigned to certain parts of the surface it should be noted that we do not expect to observe metallic Li or metallic Cu. It is well‐known that metallic Li forms a so‐called solid electrolyte interphase (SEI) layer when it comes into contact with the electrolyte.[^46^, ^47^, ^48^] Also for Cu surfaces it has been reported that a similar layer is formed upon contact with the electrolyte.^[49]^

**Table 1:** Surface roughness of the AFM topographical maps presented in Figure 3, calculated as root mean square of the height over the entire AFM map.

Position	Roughness [μm]
square	1.0
star	1.3
triangle	0.2
circle	0.3

In addition to topographical images, stiffness maps were recorded to gain a deeper insight into the mechanical properties of the species present on the sample surface. The stiffness maps at positions square and star, presented in Figure 3e and f, respectively, display a rather uniform stiffness across the surface in a range between 0 and 3 GPa, where the stiffness is mostly below 2 GPa. There are slight variations of stiffness especially on the larger protruding particles at position star. The stiffness map at position triangle depicts a rather uniform stiffness of the surface around 1 to 2 GPa. The particles which are visible in the topographic map exhibit a higher stiffness of about 3 GPa. The stiffness map at position circle, however, displays vastly different stiffness values and is hence plotted with a different colour scale. In areas where the smooth surface film is observed in the topographic image, the stiffness is rather low (below 5 GPa), similar to the stiffness observed at the other positions. The granular part of the surface that is not covered by a surface layer, however, displays a distinctively higher stiffness, which varies in a wide range up to 200 GPa. This difference in stiffness demonstrates that the smooth film and the granular surface are mechanically different species. It is assumed that the granular surface is the bare Cu foil, whereas the smooth film is introduced by Li deposition.

**Figure 4:**
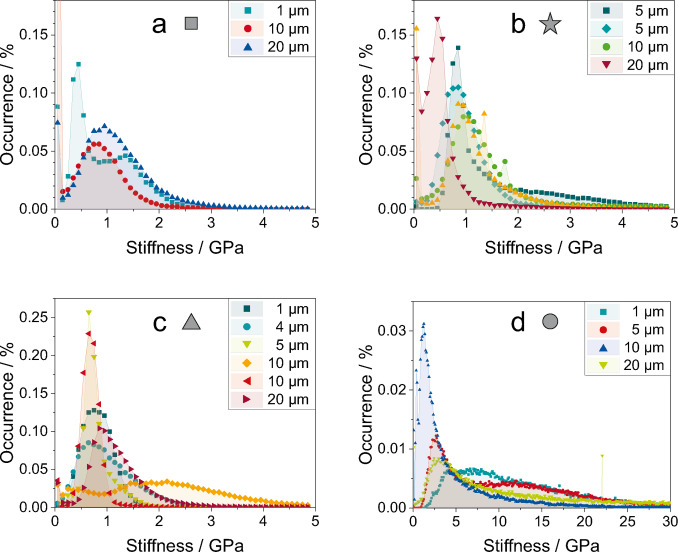
Histograms of stiffness maps obtained at different positions on the sample. Positions are marked by symbols, please refer to Figure 2. Stiffness maps were obtained in different sizes on the surface, these are represented by differently coloured symbols in the individual plots. The side lengths of the quadratic AFM images are detailed in the plot legends. The underlying AFM images are depicted in Figures S2 and S3. Histograms were calculated with a bin width of 0.1 GPa.

To allow for a more quantitative evaluation of the stiffness data, the stiffness maps presented in Figure 3d–f as well as in Figures S2 and S3 were plotted as histograms, portraying the relative occurrence of the individual stiffness values. The stiffness histograms are depicted in Figure 4. The shape of the stiffness histograms are not Gaussian, which would be expected for a single mechanical species present on the surface. Instead, the shape of the stiffness histograms can be understood as a superposition of multiple Gaussian functions. This type of shape indicates that the surface is covered by several mechanically different species. In addition to the stiffness histograms calculated from the stiffness maps in Figure 3 with a size of 20×20 μm^2^, further stiffness maps with different sizes were considered from 1×1 μm^2^ to 20×20 μm^2^. These additional stiffness histograms are depicted in Figure 4 as well. The shapes of these stiffness histograms obtained from differently sized stiffness maps vary, but align at several peak positions. This indicates a heterogeneous surface. At different positions and sizes, different parts of the surface are recorded. Hence, the distribution of stiffness values varies. The largest variation is expected to occur for large recorded areas, since the larger area includes a greater variety of species on the surface.

As observed already in the stiffness maps, the stiffness has an overall similar range of values for the positions square, star and triangle. The stiffness histogram at position circle displays overall much higher values of stiffness with a peak at stiffness values below 2 GPa that corresponds to the stiffness observed at all of the other positions. This distribution of stiffness values shows that at position circle, the surface is only partly covered by Li deposits. The similarity of stiffness of positions square, star and triangle suggests that the surface consists of mechanically similar species at these positions regardless of the morphological differences observed in the topographical maps. This is expected as Li was deposited on the Cu surface in all cases. Also the smooth patches on the surface of position circle have a similar stiffness corresponding to Li deposits, verifying that these few patches are also caused by Li deposition. As the Li metal was deposited in electrolyte, it is expected that the Li surface is covered by a layer of reaction products of Li and electrolyte, an SEI layer.^[46]^ Hence, the depicted surface and stiffness values do not correspond to Li metal itself, but rather to the stiffness of its reaction products. This is further supported by the magnitude of stiffness values which is similar to those observed *in situ* for the SEI layer on Li metal in electrolyte.^[7]^

The correlation of the observed intensity in the EPRI maps to the morphology and stiffness detected by AFM provides a deeper understanding of the deposited Li. As suggested from the lineshape of the EPR signal, the morphology observed at high intensities of the EPRI map corresponds to a “mossy” Li morphology, as also observed by AFM. It is notable that the intensity at both positions triangle and circle is low in the EPR image, whereas the surface coverage and stiffness differ between both positions. Furthermore, the stiffness at position triangle is similar to that at positions square and star, while the intensity of the EPRI map differs considerably. This observation might be attributed to the integral properties of the methods employed. EPRI is a method that only detects paramagnetic species, in this case Li metal. AFM is a surface sensitive method which is not selective to a special material. The intensity is very low for positions circle and triangle in the EPRI maps, indicating that no Li metal is detected in these positions. However, this does not necessarily have to mean that there is no Li metal present. If the amount of the deposited Li is very low, it may be below the detection limit of EPRI. This is conceivable for position circle, where Cu is clearly visible in the optical image. Such an explanation is less likely for position triangle, where considerable amounts of Li deposits are visible in the optical image. A possible explanation might be the interaction of a multi‐layered sample, and thus different conductivities, with the electromagnetic field applied during the EPR experiment. For layered systems containing a metallic or semiconducting layer it has been shown that the EPR line shape and intensity depend on the conductivity of the layers.^[31]^ As Li deposited on Cu might be interpreted as a 2‐layered system, such effects might occur for the EPR signal of Li deposited on Cu as well, leading to a signal loss.

## Conclusions

We successfully correlated EPRI with AFM. Moreover, we demonstrated that AFM is a valuable tool to understand the origin of EPRI signals while EPRI may provide chemical information on a relatively large scale that is not accessible by AFM. AFM observed two distinct morphologies which exhibit different surface roughness. Furthermore, the local stiffness shows that the sample surface is covered by various mechanically distinguishable materials. The topography and stiffness data obtained by AFM clearly show that Li deposition occurred at all positions, although with different coverages. There is an ambiguity between Li deposits observed in AFM and Li signal intensity in EPRI. AFM as well as optical images display (sparse) coverage with Li deposits at positions where EPRI does not give a signal. As AFM does not identify elements and only shows a surface layer that can be mechanically compared to an SEI layer, it cannot be verified that Li metal is present. Only the presence of Li reaction products on the surface can be confirmed. This circumstance leads to two possible conclusions. First, there may be no Li metal present at the positions with low intensity in EPRI because it reacted with either the electrolyte or trace atmospheric impurities of oxygen or water. The other explanation would be that EPRI has a limit of sensitivity and can detect predominantly mossy or dendritic Li, but has difficulties detecting bulk Li.

Considering the sensitivity limit of EPRI, it is speculated that the EPR signal is affected by the quality of contact between Li and Cu, which could lead to a complete signal loss for a thin Li layer in very good contact with Cu. The oscillating electromagnetic field used to excite the electron spins experiences a multi‐layer system with different conductivities and, therefore, different impedances. If the contact between the Li and the Cu layer is poor, conduction electrons are less likely to pass the interface, and the conduction EPR of Li metal can be described by the theory of Dyson.^[27]^ However, if a thin Li layer is in very good contact with Cu, an electron transfer across the interface leads to almost instantaneous electron spin relaxation, leading to line broadening or, for very thin Li layers, to complete loss of the conduction EPR signal of Li metal. This presents an intriguing additional aspect for the correlation of EPRI, optical images and AFM to probe the quality of contact between Li and Cu. For a bad contact, a longer relaxation time would be expected, leading to narrower lines, as observed for isolated layers of Li metal. For a good contact, the EPR signal of thin Li layers gets reduced or might even be suppressed completely. An example for such an effect can be seen by comparing Figures 2b and c. Near the region where the AFM data was taken, a small piece of Li has broken off, exposing shiny Cu metal underneath. This indicates poor electrical contact between the two layers. The corresponding EPRI signal, which was recorded before the piece broke off, shows the expected high signal intensity. On the other hand, position triangle and, even more pronounced, the region below position star, show no substantial signal in the EPRI map, which would be consistent with a very good contact between the two layers. However, a deeper investigation is beyond the scope of this study. In subsequent studies, spectral‐spatial imaging could help in studying different types of Li species. This technique provides a spatial resolution of the EPR spectrum in addition to the EPRI intensity, yet protocols need to be developed specifically for conduction EPRI.^[28]^ Furthermore, the recently proposed method of second harmonic detection of EPRI could be a simpler tool to distinguish Li metal species.^[50]^

## Conflict of Interests

There are no conflicts of interest to declare.

1

## Supporting information

As a service to our authors and readers, this journal provides supporting information supplied by the authors. Such materials are peer reviewed and may be re‐organized for online delivery, but are not copy‐edited or typeset. Technical support issues arising from supporting information (other than missing files) should be addressed to the authors.

Supporting Information

## Data Availability

The data that support the findings of this study are available from the corresponding author upon reasonable request.
