# Supplementary material for: Correlative Electron Paramagnetic Resonance Imaging and Atomic Force Microscopy of Lithium Deposited on Copper
Source: Chemphyschem. 2025 Jan 24;26(6):e202400937. doi: 10.1002/cphc.202400937 (PMC11913469; doi:10.1002/cphc.202400937)
Supplement: Supplementary file 1 — Supporting Information [file CPHC-26-e202400937-s001.pdf]

ChemPhysChem

Supporting Information

Correlative Electron Paramagnetic Resonance Imaging and Atomic Force Microscopy of Lithium Deposited on Copper

Beatrice Wolff, Christian Hellenbrandt, Peter Jakes, Rüdiger-A. Eichel, Josef Granwehr,* and Florian Hausen*

Supporting Information

Correlative Electron Paramagnetic Resonance Imaging and Atomic Force Microscopy of Lithium Deposited on Copper

Beatrice Wolff,[a,b] Christian Hellenbrandt,[a] Peter Jakes,[a] Rüdiger-A. Eichel,[a,b] Josef Granwehr*,[a,c] Florian Hausen*[a,b]

[a] B. Wolff, C. Hellenbrandt, Dr. P. Jakes, Prof. Dr. R.-A. Eichel, Prof. Dr. J. Granwehr*, Prof. Dr. F. Hausen*
Forschungszentrum Jülich GmbH, Institute of Energy Technologies, IET-1, 52425 Jülich, Germany
E-mail: f.hausen@fz-juelich.de; j.granwehr@fz-juelich.de

[b] B. Wolff, Prof. Dr. R.-A. Eichel, Prof. Dr. F. Hausen*
RWTH Aachen University, Institute of Physical Chemistry, 52074 Aachen, Germany

[c] Prof. Dr. J. Granwehr*
RWTH Aachen University, Institute of Technical and Macromolecular Chemistry, 52074 Aachen, Germany

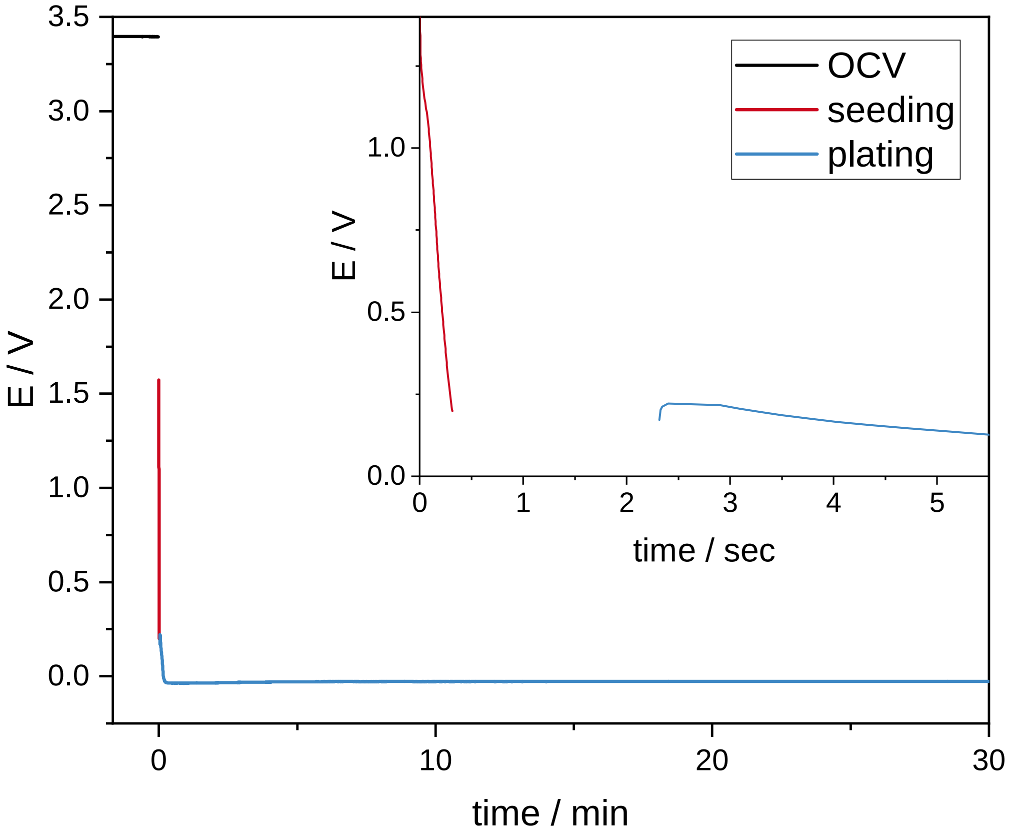

Figure S1: Voltage profile of Li deposition on Cu during sample preparation. The black line refers to the open cirucuit voltage (OCV) before Li was deposited. A seeding step (red line) was applied for 0.3 s at a current density of -20 mA/cm^2. Afterwards, Li was plated at a current density of -1 mA/cm^2 for 30 min (blue line).

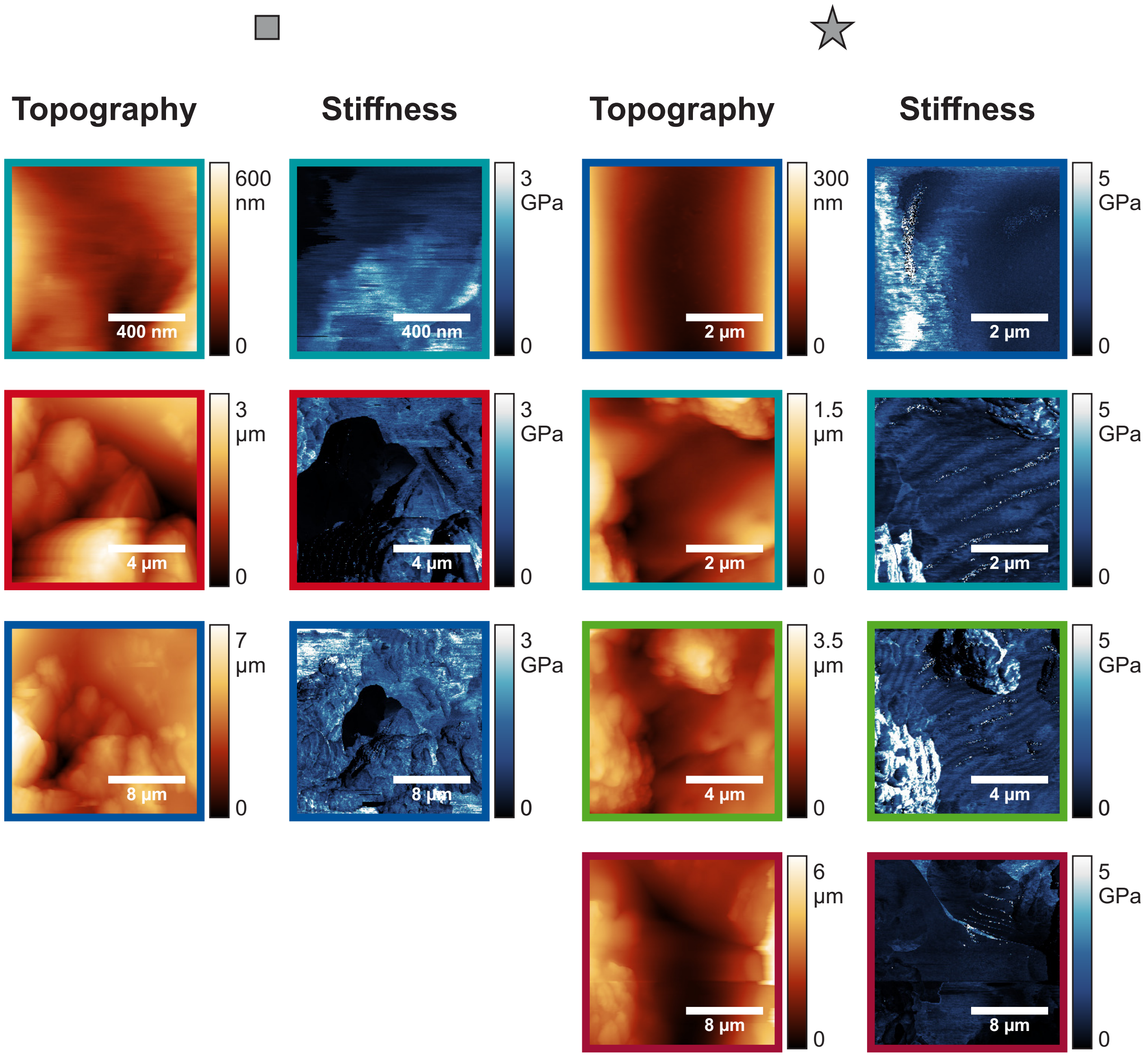

Figure S2: Topography and stiffness maps of the stiffness histograms presented in Figure 4a and b for positions square and star, respectively. The coloured frames correspond to the colours of the respective symbols in the histogram plots.

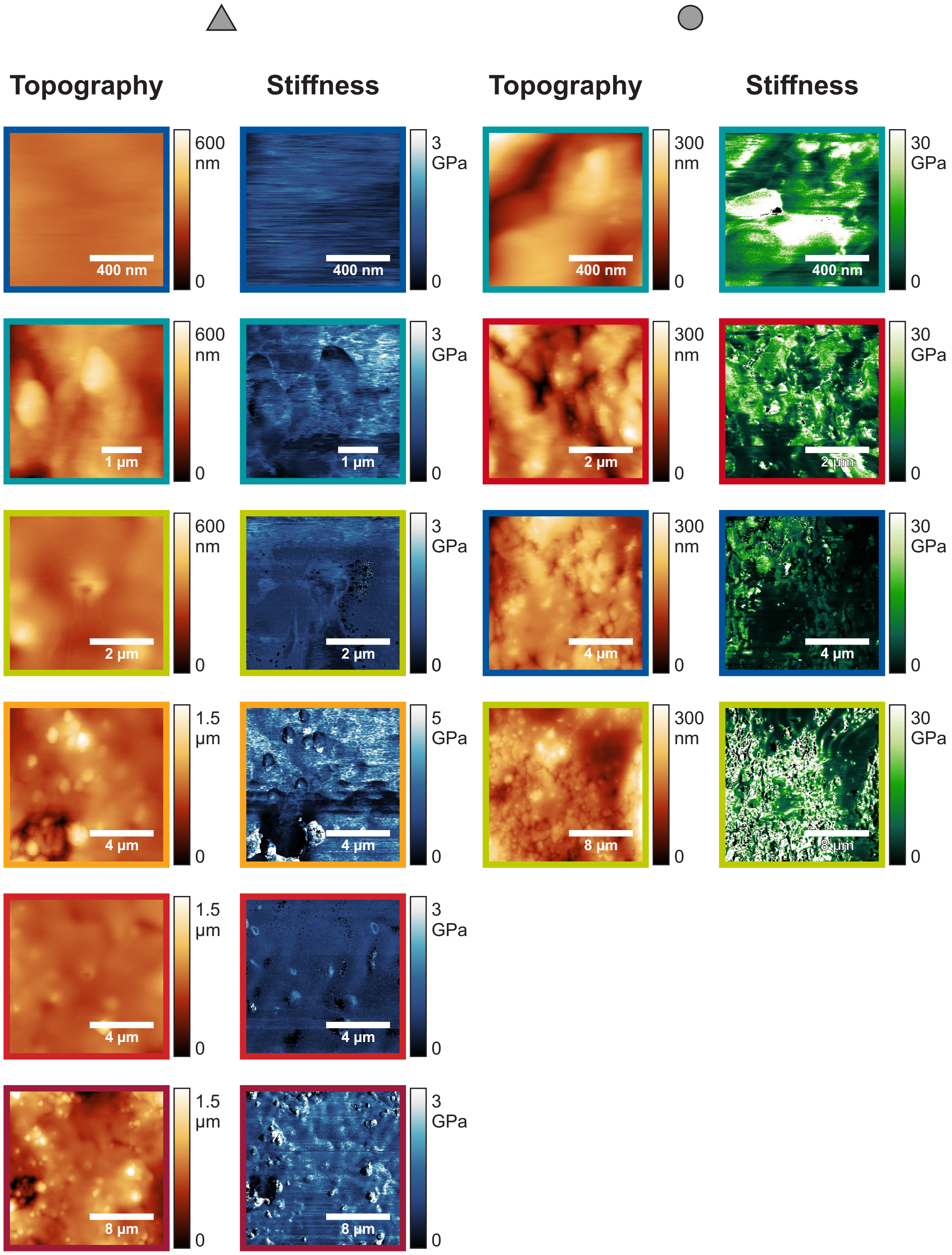

Figure S3: Topography and stiffness maps of the stiffness histograms presented in Figure 4c and d for positions triangle and circle, respectively. The coloured frames correspond to the colours of the respective symbols in the histogram plots.
